# Analysis of non-volatile and volatile metabolites reveals the influence of second-drying heat transfer methods on green tea quality

**DOI:** 10.1016/j.fochx.2022.100354

**Published:** 2022-06-03

**Authors:** Huajie Wang, Wen Ouyang, Yaya Yu, Jinjin Wang, Haibo Yuan, Jinjie Hua, Yongwen Jiang

**Affiliations:** a Tea Research Institute, Chinese Academy of Agricultural Sciences, 9 Meiling South Road, Hangzhou, Zhejiang 310008, PR China; b Institute of Tea Science, Zhejiang University, Hangzhou 310058, PR China; c State Key Laboratory of Tea Plant Biology and Utilization, Anhui Agricultural University, Hefei 230036, Anhui, PR China

**Keywords:** Green tea, Heat transfer methods, Second-drying, Amino acids, Chlorophyll, Odor activity value, FISD, far-infrared second-drying, MWSD, microwave second-drying, BASD, box-hot air second-drying, CMSD, carding machine second-drying, RPSD, rotary pot second-drying, IRAE-HS-SPME, infrared-assisted coupled to headspace solid-phase microextraction, MEV, Multiple experiment viewer, PLS-DA, Partial least-squares discriminant analysis, OAV, Odor activity value, GC-MS, Gas chromatography-tandem mass spectrometry

## Abstract

Second-drying is a key process of green tea manufacturing, however, hitherto the effect of second-drying methods on green tea quality has not been assessed. In this study, we compared the effect of three heat transfer drying methods (heat radiation, heat convection, and heat conduction) on green tea quality. Gas chromatography-tandem dual mass spectrometry was used to detect volatile compounds, while absolute quantitative methods were used to detect the non-volatile ones. We identified 45 non-volatile metabolites, 101 volatile metabolites, and 15 objective flavor indicators. Seventeen differential non-volatiles and 8 differential volatiles were screened. Microwave second-drying in heat radiation was the optimal method for green tea flavor, as it can promote the retention of chlorophyll, the degradation of flavonoid glycosides, and the enrichment of amino acids, soluble sugars, nonanal, *trans*-β-ionone, linalool, and jasmone. The results provide a theoretical basis and technical guidance for the precise and directional processing of high-quality green tea.

## Highlights

• Effect of second-drying heat-transfer modes on green tea quality was investigated.
• Microwave second-drying (MWSD) was the optimal method for green tea aroma and color.
• Seventeen non-volatile and eight volatile differential metabolites were identified.
• Nonanal, *trans*-β-ionone, linalool, and jasmone had highest content in MWSD.
• MWSD was beneficial to the retention of chlorophyll, theanine, and soluble sugars.

## Graphical abstract

**Figure:**
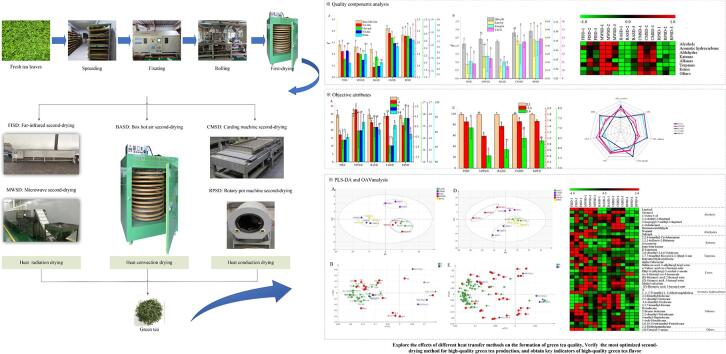

## Introduction

Green tea, with the largest sales volume, accounts for approximately 60% of total tea output in China (Liu et al., 2021). Green tea is becoming increasingly popular among consumers owing to its green color, brisk and mellow taste, strong aroma, and antioxidant, anti-cancer, anti-radiation, antibacterial, and other health-promoting properties (Hajiaghaalipour et al., 2016, Liu et al., 2021, Cheng et al., 2020, Mohammad et al., 2021, Daniel, 2021). The quality of green tea is most closely related to the processing technology. The initial processing of green tea generally involves spreading, fixation, rolling, first-drying, and second-drying.

The second-drying step is a decisive process for the formation of green tea color, aroma, taste, and appearance. Second-drying promotes water loss, which facilitates storage and transport and promotes the formation of an excellent shape. Moreover, second-drying further promotes the low boiling point volatile metabolites with grass aroma in tea leaves under the action of high-temperature loss, and greatly increases the compounds with floral and roasted aromas. Second-drying methods can be divided into three major categories: heat radiation, heat convection, and heat conduction (Wang et al., 2020a), according to the difference in heat conductivity of the media (wave energy, air, and metal). At present, the second-drying methods used in tea processing mainly include chain plates, box-hot air, rollers, carding machines, far-infrared, and microwaves (Chen et al., 2014, Wang et al., 2014, Liu et al., 2015, Qu et al., 2019, Wang et al., 2021).

By comparing the effects of different drying methods on the sensory quality of green tea, it was found that roller drying and box-hot air drying are beneficial for the formation of the chestnut-like aroma, while microwave and far-infrared drying are more beneficial for the formation of tender fragrances (Liu et al., 2012, Chen et al., 2014, Liu et al., 2015a, Zhang et al., 2020). That is, diverse drying methods with different heat transfer characteristics result in the unique flavor quality of green tea. A number of studies have proved that microwave drying can rapidly increase the temperature of the tea samples, through the effect of high-frequency microwave oscillation. Furthermore, microwave drying can quickly fix the quality of green tea, which is conducive to the retention of chlorophyll, and promote the green appearance of leaves, green soup, and green infused leaves (Wu et al., 2021, Guo et al., 2016, Yuan et al., 2009). However, in previous studies. Only two or three drying methods have been selected as objects for comparison. The quality evaluation of tea samples is mostly based on sensory evaluation and some quality components, which are not objective or comprehensive. In addition, from the aspect of the heat transfer mechanism, the influence of second-drying methods on the quality of green tea has not been assessed to date.

Therefore, this study selected three types of heat transfer methods that are widely used in the processing industry: heat radiation (far-infrared second-drying; FISD, microwave second-drying; MWSD), heat convection (box-hot air second-drying; BASD), and heat conduction (carding machine second-drying; CMSD, rotary pot second-drying; RPSD) to make green tea. Important non-volatile metabolites (i.e., catechin components, flavonoid glycoside components, amino acid components, tea polyphenols (TPs), and soluble sugars) were detected with absolute quantitative methods. Volatile metabolites were detected semi-quantitatively with infrared-assisted headspace solid-phase microextraction (IRAE-HS-SPME) and gas chromatography-mass spectrometry (GC-MS). Simultaneously, the objective flavor indexes (i.e., appearance, soup, and taste attributes) were analyzed in combination with expert sensory evaluation. Moreover, partial least square discriminant analysis (PLS-DA) and odor activity value (OAV) analyses were used to clarify the effect of heat transfer methods on the quality of green tea, to identify the key differential metabolites, and to obtain an optimized second-drying method for high-quality green tea. This study enriches the basic theory of green tea processing chemistry, and provides theoretical support and technical guidance for the standardization and directional processing of high-quality green teas.

## Materials and methods

### Experimental materials

One bud and one leave of “CuiFeng” were selected in this study, and picked in April 2020 in KaiHua (Zhejiang, China).

All the chromatography-grade standards, such as catechins, amino acids, flavonoid glycosides, gallic acid, and caffeine were purchased from Shanghai Yuanye Biological Technology Company (Shanghai, China). The purity of these chemical standards was ≥ 98%.

### Green tea sample preparation

**Fig. 1:**
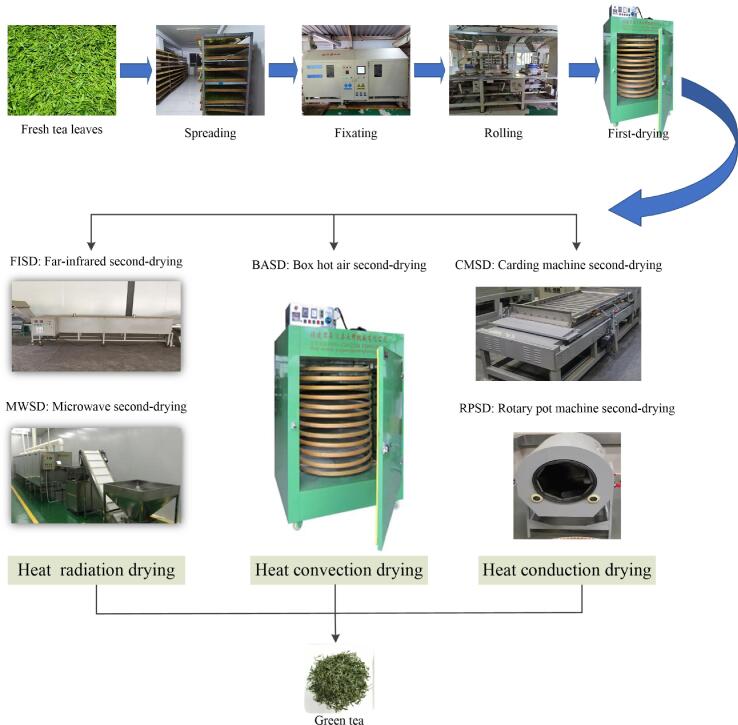
Green tea processing flow chart. (For interpretation of the references to color in this figure legend, the reader is referred to the web version of this article.)

Tea making process is shown in Fig. 1:

1) Spreading. Tea leaves were spread and kept for 12 h in a YJY-20 M type withering machine (Yuyao Yaojiangyuan Tea Maker Co., Ltd., Ningbo, China) at a temperature of 20.0 ± 1.0 °C with a relative humidity of 58 ± 3.0% until the moisture of tea leaves reached 70%.
2) Fixating. Spread tea leaves were fixed for 150 s in a 6CSF-80 type roller-hot air fixation machine (Yuyao Yaojiangyuan Tea Maker Co., Ltd., Ningbo, China) with a rolling speed of 22 rpm, a rolling temperature of 270 °C + 250 °C + 230 °C, and 150 kg of tea leaves per hour.
3) Rolling. The fixed leaves were rolled for 35 min in a 6CR-45 type rolling machine (Zhejiang Shangyang Machinery Co., Ltd., Zhejiang, China).
4) First-drying. The rolled samples were first dried for 15 min at 110 °C, until moisture content of leaves up to 20%∼25%.
5) Second-drying. The first-dried leaves were treated in three different heat transfer methods to obtain green tea with a moisture content of approximately 6%.

Heat radiation drying: FISD, the first-dried tea samples were spread on a far-infrared drying machine (DXCWS-05 Yixing Dingxin microwave Equipment Co., Ltd., Jiangsu, China) and subjected to drying at 115 °C for 6 min. MWSD, the first-dried tea samples were spread on a microwave drying machine (Jiangsu Agricultural Mechanization Research Institute Jiangsu, China) and dried at 115 °C for 5 min.

Heat convection drying: BASD, the first-dried tea leaves were spread in box-hot air drying machine (JY-6CHZ-7B, Fujian Jiayou Machinery Co., Ltd., Fujian, China) and dried at 90 °C for 30 min.

Heat conduction drying: CMSD, the first-dried tea leaves were dried in a carding drying machine (6CMD-6018, Zhejiang Lvfeng Machinery Co., Ltd., Zhejiang, China) and dried at 150 °C for 20 min; RPSD, the first-dried leaves were dried in a rotary pot machine (6CMG-63A, Zhejiang Xinchang Silver Ball Machinery Co., Ltd., Zhejiang, China) and dried at 150 °C for 10 min.

### Analyses of non-volatile metabolites in green tea

#### Detection of TPs, gallic acid (GA), and caffeine (CAF)

The contents of total polyphenols were detected by “Folin Reagent Colorimetry (GB/T 8313_2008)”; the contents of CAF, GA, and catechins were characterized using high-performance liquid chromatography (Wang et al., 2022, Hua et al., 2020, Blaženović et al., 2018, Sumner et al., 2007) (HPLC, Agilent 1100 V L, Agilent Technologies Inc., CA, USA).

#### Detection of total amino acids and amino acids components, soluble sugars, chlorophyll

The content of total aminos was characterized using “Ninhydrin colorimetry (GB/T 8314-2002)”, amino acid composition was detected using the automatic amino acid analyzer method (Hua et al., 2021), and soluble sugars were detected using the “Anthrone colorimetry” method (Wang et al., 2020a). After pulverizing the tea with a high-speed pulverizer, chlorophyll was passed through a 100-mesh sieve and measured with a chlorophyll kit from the Nanjing Jiancheng Institute of Biological Engineering (Wang et al., 2019).

#### Detection of flavonoid glycoside components

Chromatographic column: C18 column (4.6 mm × 250 mm, 5 μm); mobile phase A phase: 0.15% formic acid aqueous solution; B phase: acetonitrile. Mobile phase elution gradient: 0 ∼ 2 min, 6%∼17%B; 2 ∼ 22 min, 17%∼19%B; 22 ∼ 23 min, 19%∼30%; 23 ∼ 25 min, 30%∼6%; 26 ∼ 30 min, 6%. The flow rate was 1 mL/min, the column temperature was 35 °C, the injection volume was 20 μL, and the detection wavelength was 360 nm (Blaženović et al., 2018, Sumner et al., 2007, Wang et al., 2020a).

### Analysis of volatile metabolites in green tea

#### Sample pretreatment

Tea samples (0.5 g), 1 mL of boiling distilled water, and 10 μL (20 mg/L) of ethyl caprate (chromatographic grade; TCI Chemical Industry Development Co. Ltd., Shanghai, China) were added in a 20 mL headspace vial. Then immediately inserted into the headspace vial using the manual handle (SPME, Supelco, USA) with the DVB/CAR/PDMS fiber head (Supelco). The vial was placed on a 100 w infrared device (Qiyi Lighting Company, Zhejiang, China) unit for 15 min, then the fiber head was inserted into the GC-MS for 5 min, and each sample was repeated three times (Yang et al., 2020).

#### GC-MS/MS analysis

Agilent Technologies 7890B-7000C was used to detect volatile metabolites. The ultra-inert capillary column was HP-5 MS (30 m × 0.25 mm × 0.25 μm) with high-purity helium (99.999%) and a flow rate of 1.0 mL/min. The temperature of the GC injector was maintained at 250 °C, the column temperature program was as follows: 50 °C, 5 min, increased to 150 °C at 4 °C/min and maintained for 2 min, increased to 270 °C at 10 °C/min and maintained for 6 min (Wang et al., 2020b).

Qualitative analysis was processed with Agilent MassHunter Workstation Software Unknowns Analysis according to the principle of over 80% similarity to the NIST library. Kovàts retention indices for each compound were obtained by calculating the linear formula of *n*-alkanes (C7-C40) and by comparing with the theoretical retention index. The concentration of volatile compounds was calculated with reference to the internal standards (Blaženović et al., 2018, Sumner et al., 2007, Wang et al., 2020b).

#### Odor activity value

OAV=C/OT

C is the concentration of the volatile metabolite (μg/L); OT is the metabolite odor threshold (values in water, μg/L) (Yang et al., 2021).

### Sensory evaluation

“Methodology of sensory evaluation of tea” (GB/T 23776-2018) was used to assess green tea (Wang et al., 2020c). A quality sensory panel group (three men, two women, aged between 28 and 48 years old) took 50 g of tea samples to the sample tray. First, they assessed the appearance, then weighed 3 g of tea sample, placed it in a porcelain cup, added 150 mL of boiling water, and brewed it for 5 min. They then conducted a code review. The tea quality was assessed using a combination of comments and 100-point scoring, including appearance, aroma, liquor color, taste, infused leaves, with 100 points for each item.

Totalsensoryscore=aromascore×25%+tastescore×30%+shapescore×25%+liquorcolorscore×10%+infusedleavesscore×10%

### Analyses of objective indicators

#### Detection of appearance and liquor color index of dry tea samples

*Appearance color index:* The color appearance of the tea samples was triangulated by a portable colorimeter (CM-600d, Konica Minolta Investment Co. Ltd., Shanghai, China). “L” represents the brightness; “a” represents the degree of red-green, “b” represents the degree of yellow-blue, “c” represents saturation; “h” represents hue angle (Hua et al., 2020).

*Liquor color index:* a Konica Minolta tabletop spectrophotometer (CM-5 type, Shanghai, China) was used to measure the value of LL, La, and Lb of the tea liquor. “LL” represents the translucent degree, “LL” represents red-green degree, and “Lb” represents yellow-blue degree (Hua et al., 2020).

#### Analysis of green tea taste

The tea samples were brewed using the method in section *2.5*, and the tea broth was poured into a glass beaker while hot through 4 layers of gauze (150 mesh × 10 mesh) and cooled to room temperature (25 ± 2 ℃). Each tea sample was brewed in triplicate. Seven tea broth replicates were performed, and the stable measurement data of the last three replicates were selected for subsequent analysis (Yao et al., 2019).

### Statistical analysis

The tests were repeated in triplicate and the data from each test were expressed as the average of the triplicate. A one-way ANOVA was used to show data significance (*p* < 0.05) using SAS software (version 9.4; SAS Institute Inc., Cary, NC, USA). The SIMCA-P13 software (Umetrics, Umea, Sweden) was used to analyze the principal components and obtain key differences in non-volatile and volatile metabolites under different drying methods. Origin 2018 software (Origin Lab, USA) was used to analyze metabolite differences under different drying methods. MEV 9.0 software (https://mev-tm4-org.caas.cn) was used to compare the contribution of amino acid and volatile metabolites under processing with different drying methods.

## Results and discussion

### Effect of heat transfer methods on the content of non-volatile quality components in green tea

#### TPs, catechin components, GA, and CAF

TPs, catechins, GA, and CAF are important components of green tea taste (Xu et al., 2019). The TPs content varied significantly (*p* < 0.05) under the different second-drying methods (Table 1) and was significantly highest (*p* < 0.05) in the CMSD, followed by the RPSD and the FISD. The TPs content of the MWSD and BASD were significantly lower (*p* < 0.05) than that of CMSD. The total amount of catechins (TAC), total ester-type catechins (TETC), and total amount of simple catechins (TSC) showed a trend of RPSD > CMSD > FISD > BASD > MWSD and the six catechin components were also significantly higher in RPSD (*p* < 0.05), followed by CMSD. The TSC/TETC value was significantly higher in BASD, followed by FISD and MWSD. The content of GA showed a changing trend of RPSD, CMSD > FISD, BASD > MWSD; CAF, as the main bitter taste contributor, also had a strengthening effect on the astringency of catechins and its content in RPSD and CMSD was significantly higher than that in the others (*p* < 0.05).

**Table 1:** Analysis table of polyphenols, catechin components and total content, gallic acid, caffeine, and chlorophylls in green tea under different second-drying heat transfer methods.

Compounds (%)	FISD	MWSD	BASD	CMSD	RPSD
TPs	11.292 ± 0.58b	10.261 ± 0.392c	10.196 ± 0.308c	12.701 ± 0.301a	11.912 ± 0.182b
GC	0.198 ± 0.01b	0.124 ± 0.011d	0.175 ± c	0.216 ± 0.008ab	0.23 ± 0.012a
EGC	0.62 ± 0.035b	0.608 ± 0.051b	0.604 ± 0.046b	0.776 ± 0.035a	0.848 ± 0.059a
C	1.084 ± 0.014c	1.067 ± 0.038c	1.135 ± 0.05c	1.435 ± 0.055b	1.527 ± 0.052a
EGCG	4.65 ± 0.178c	4.51 ± 0.185c	4.547 ± 0.18c	6.247 ± 0.136b	6.674 ± 0.16a
GCG	0.123 ± 0.005b	0.088 ± 0.005c	0.089 ± 0.002c	0.13 ± 0.004b	0.144 ± 0.004a
ECG	0.948 ± 0.048b	0.906 ± 0.052b	0.904 ± 0.049b	1.313 ± 0.05a	1.383 ± 0.05a
TETC	5.722 ± 0.231c	5.505 ± 0.241c	5.541 ± 0.231c	7.69 ± 0.19b	8.2 ± 0.214a
TSC	1.901 ± cd	1.799 ± 0.099d	1.913 ± 0.105c	2.426 ± 0.098b	2.606 ± 0.123a
TSC/TETC	0.332 ± 0.008ab	0.327 ± 0.014ab	0.345 ± 0.015a	0.316 ± 0.017b	0.318 ± 0.019b
TAC	7.623 ± 0.289c	7.304 ± 0.34c	7.454 ± 0.336c	10.116 ± 0.288b	10.806 ± 0.337a
GA	0.846 ± 0.009b	0.763 ± 0.006c	0.832 ± 0.025b	0.979 ± 0.04a	1.011 ± 0.012a
CAF	1.796 ± 0.035c	1.824 ± 0.023bc	1.87 ± 0.05b	2.01 ± 0.029a	2.029 ± 0.037a
Chlorophyll *a*	0.93 ± 0.01c	1.03 ± 0.02a	0.99 ± 0.01b	1.04 ± 0.02a	0.96 ± 0.03bc
Chlorophyll *b*	0.25 ± 0c	0.32 ± 0.01a	0.33 ± 0.02a	0.33 ± 0.01a	0.29 ± 0.01b
Chlorophyll (a + b)	1.18 ± 0.01d	1.35 ± 0.03ab	1.32 ± 0b	1.36 ± 0.01a	1.25 ± 0.04c

Notes: The capital letter represents the difference in tea sample contents in different drying methods at a level of 0.05.

The changes in the contents of TPs, catechins, GA, and CAF were affected by the synergistic effect of temperature and time of different heat transfer modes. The heat conduction mode (CMSD, RPSD) caused the tea sample to continuously contact and rub the high-temperature (150 ℃) pot wall, resulting in a greater rate of cell damage which in turn led to the extraction of tea polyphenols, catechins, GA, and CAF. Heat convection drying at a low temperature over a long period was not conducive to the transformation and accumulation of quality substances. Appropriate reduction of TPs and catechins decreases the phenol-amino ratio to promote a fresh taste without bitterness (Wan, 2003), conversely, the tea soup would be more bitter and astringent if the ratio is too high. otherwise, the tea soup will be light and tasteless.

#### Chlorophyll

Chlorophyll plays an important role in the appearance and liquor color of green tea (Li et al., 2021). During processing, chlorophyll can undergo a series of transformations (isomerization and demetallation) triggered by chlorophyllase-mediated or non-enzyme-mediated thermal reactions (Wu et al., 2021). Different second-drying methods had significant effects on the chlorophyll content (Table 1). Chlorophyll *a* was significantly higher in CMSD and MWSD, followed by BASD and RPSD, and was significantly lowest in FISD (*p* < 0.05). Chlorophyll *b* was significantly higher in CMSD, BASD, and MWSD than in FISD and RPSD (*p* < 0.05). Chlorophyll (a + b) showed a trend of CMSD > MWSD > BASD > RPSD > FISD, namely the degree of isomerization reaction, demetallation reaction, and decarboxymethylation reaction was lowest under MWSD, which is consistent with previous studies (Wu et al., 2021). However, the trend of chlorophyll content in the second-drying carding machine is inconsistent with previous studies (Wu et al., 2021), which is speculated to be related to the different settings of CMSD parameters.

The high-frequency vibration of MWSD promotes the high-speed collision of the internal molecules to generate a large amount of heat, rapidly increase the temperature of tea leaves, result in a large amount of chlorophyll retention, and prevent isomerization and demetallation (Wan, 2003). Far-infrared secondary drying causes the tea leaves to rapidly heat up and lose water. Critically, chlorophyll is easily degraded under high-temperature roasting. Forty minutes of heat convection drying (BASD) promoted the conversion and loss of chlorophyll. The long duration and high-temperature conditions of RPSD promoted the degradation of chlorophyll in a relatively confined space, while the retention of chlorophyll was higher under CMSD in a relatively open space. In RPSD, the relative humidity of second-drying significantly affected chlorophyll content, whereas, in CMSD, the low relative humidity condition promoted the retention of chlorophyll (Zhang et al., 2020).

#### Flavonoid glycosides

**Fig. 2:**
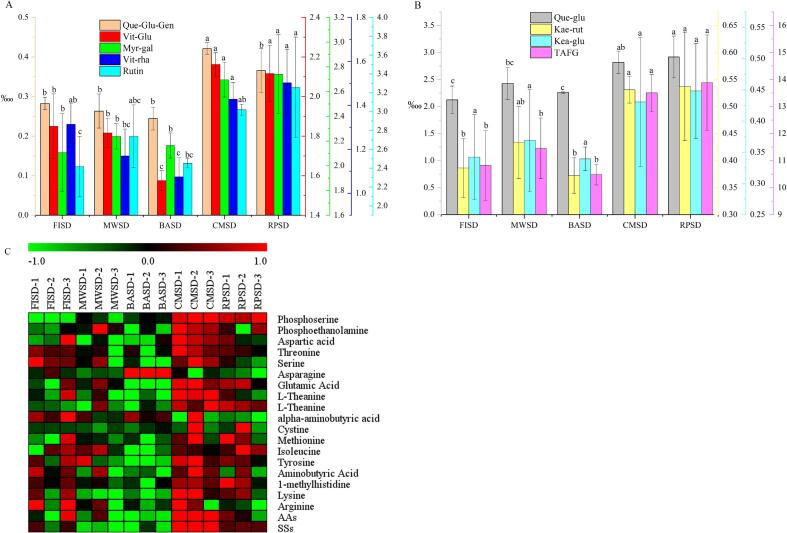
(A-B) Column plot of flavonoid glycosides components in green tea under different second-drying heat transfer methods. (C) Thermography of amino acid composition and soluble sugars in green tea under different second-drying heat transfer methods. Note: Que-Glu-Gen, Quercetin 3-O-β-d-glucose-7-O-β-d-gentiobioside; Vit-Glu, Glucosyl-vitexin; Myr-gal, Myricetin 3-O-galactoside, Vit-rha, Vitexin-2-O-rhamnoside, Que-glu, Quercetin-3-O-glucuronide, Kea-rut, Kaempferol-3-Rutinoside, Kea-glu, kaempferol 3-O-glucoside, TAFG, Total flavonoid glycosides; AAs, Total amino acids; SSs, Total soluble sugars. (For interpretation of the references to color in this figure legend, the reader is referred to the web version of this article.)

Flavonoid glycosides are not only related to liquor color but are also important contributors to the taste of green tea. The heat transfer methods had a significant effect on the flavonoid glycoside (Fig. 2A–C). Eight flavonoid glycoside components were highest in CMSD and RPSD (*p* < 0.05). The total amount of flavonoid glycosides after second-drying showed a trend of: RPSD (13.885%) > CMSD (13.507%) > MWSD (11.456%) > FISD (10.823%) > BASD (10.485%). That is, compared with heat-conduction drying, the difference in flavonoid glycoside content under heat radiation and heat convection was smaller. Due to the direct contact between the leaves and the heat source under the heat conduction method, the leaf tissues became damaged and bound flavonoid glycosides were hydrolyzed, inducing a high content of flavonoid glycosides. This result is consistent with that of a previous study on black tea (Wang et al., 2020a). That is, different heat transfer modes lead to distinctive rates of cell breakage and material leaching, resulting in differences in the content of quality components and, finally, green tea with different flavor qualities.

#### Amino acids and soluble sugars

Amino acids are important substrates for the fresh and brisk tastes of green tea (Wang et al., 2016, Zhang et al., 2015, Ho et al., 2015). As shown in Fig. 2D, the content of each amino acid component (except asparagine and γ-aminobutyric acid) was significantly higher under CMSD and RPSD (*p* < 0.05), followed by FISD and MWSD, whereas BASD had the lowest. The main amino acid components, such as l-theanine, glutamic acid, arginine, lysine, and isoleucine, followed the same trend: CMSD > RPSD > FISD > MWSD > BASD. Soluble sugar is an important basic substance in the sweet taste and aroma of green tea (Ho et al., 2015, Wang et al., 2020a). Sugar was significantly highest in CMSD and RPSD (*p* < 0.05), where sugar content was above 1.65%.

Heat conduction contributes to the leaching and the retention of soluble sugars and amino acids. Under heat radiation, with short-term treatment, the leaf cells remained relatively intact, and the contents of amino acid components and soluble sugars were moderate. Heat convection, with the longest drying time, has a low material leaching rate and excessive thermal reaction, resulting in the lowest amino acid content.

### Effects of heat transfer methods on the content of volatile metabolites in green tea

**Fig. 3:**
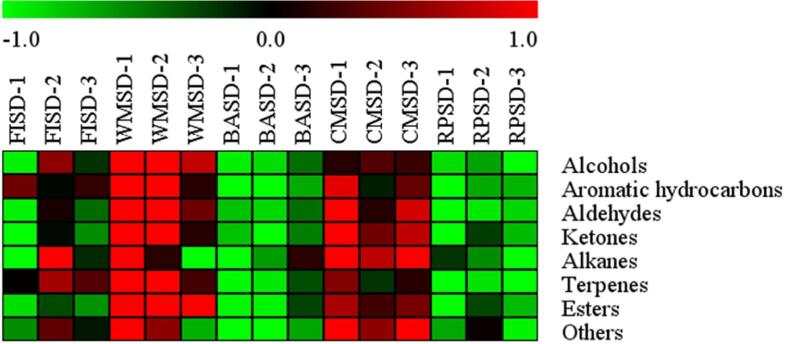
Heat map of levels of volatile category metabolites in green tea under different second-drying heat transfer methods. (For interpretation of the references to color in this figure legend, the reader is referred to the web version of this article.)

In total, 101 volatile metabolites were identified (Table S1), including 14 alcohols, seven aldehydes, 14 esters, seven ketones, 14 terpenes, nine aromatic hydrocarbons, 32 alkanes, and three other compounds. Among them, the alcohols, esters, ketones, alkenes, and aromatic hydrocarbons contents were higher than the other compounds. As shown in Fig. 3, the total amount of volatile metabolites after second-drying showed the following trend: MWSD > CMSD > FISD > BASD > RPSD. Among them, aldehydes, ketones, and esters were significantly higher in MWSD and CMSD than in the other treatments (*p* < 0.05); alcohols, terpenes, and aromatic hydrocarbons were significantly higher in MWSD (*p* < 0.05), followed by CMSD and FISD (*p* < 0.05). Alkanes and others were significantly higher in CMSD, compared to other treatments.

Microwave and carding machine second-drying can effectively retain and form high-boiling-point substances. In the heat radiation mode, tea leaves can be heated uniformly and quickly in MWSD, which can fix the quality components effectively and is consistent with existing research (Hua et al., 2020). In the heat conduction mode, the friction of tea leaves with CMSD continuously led to an increase in the cell fragmentation rate, volatile compounds, and their precursor overflow, which promoted the formation of high-boiling point volatile metabolites. The content of volatile metabolites in RPSD was lower than that in CMSD with the same heat temperature because RPSD was heated for a shorter time. The heat convection mode, with a low rate of leaf cell breakage and long-term heating, caused the formation and retention of fewer high-boiling metabolites.

### Effects of heat transfer methods on sensory quality and objective flavor indexes of green tea

#### Sensory quality

The effects of the different heat transfer methods on the sensory quality of green tea are shown in Table 2. In terms of appearance, MWSD, with a strong bud and leaves, tippy, and yellow-green color, was considered the best while FISD was considered the worst. In terms of liquor, RPSD, with the yellow-green liquor color, was considered the best. MWSD showed a tender and flowery aroma and obtained the highest score; BASD, RPSD, and FISD achieved a chestnut-like aroma, while FISD showed a slightly green and pure aroma. MWSD has the most optimal strong and mellow taste, followed by BASD, CMSD, and RPSD. Overall, the total score showed a trend of MWSD > BASD > RPSD > CMSD > FISD; that is, the sensory quality of green tea produced by microwave drying was the best.

**Table 2:** Sensory evaluation results of green tea samples processed using various second-drying methods.

Second-drying methods	Appearance		Liquor color		Aroma		Taste		Infused leaves		Total score
	Evaluation	Score	Evaluation	Score	Evaluation	Score	Evaluation	Score	Evaluation	Score	
FISD	Barely fat and bold bud, tippy, yellow-green and slight dull	89.17 ± 0.29d	yellow	88.33 ± 0.29d	Tender, chestnut obviously	91.5 ± 0.50c	Barely strong and mellow (slight bitter)	87 ± 0.50d	Fat and bold, yellow-green and slight dull	92.33 ± 0.29ab	89.33 ± 0.13e
MWSD	Barely fat and bold bud, Tippy, yellow-green	94.5 ± 0.5a	Slight yellow	89.17 ± 0.29c	Tender, faint and flowery	93.5 ± 0.50a	Barely strong and mellow	93.17 ± 0.76a	Fat and bold, Yellow-green	92.5 ± 0.50ab	93.12 ± 0.42a
BASD	Barely fat and bold bud, tippy, yellow-green and slight dull	91 ± 0.00c	green	91.33 ± 0.29b	Chestnut	92.33 ± 0.29b	Barely umami and mellow, strong	90.17 ± 0.29b	Yellow-green	92.83 ± 0.29a	91.3 ± 0.09b
CMSD	Barely Fat and bold bud, tighter Tippy, yellow-green and glossy	92 ± 0.50b	Yellow-green	91 ± 0.00b	Barely green, pure	89 ± 0.50d	Barely strong, slight harsh	88.17 ± 0.29c	Yellow-green bright	92.83 ± 0.29a	90.08 ± 0.10d
RPSD	Barely fat and bold bud, loose Tippy, yellow-green	91.17 ± 0.29c	Slight yellow-green	92.33 ± 0.29a	chestnut	92.17 ± 0.29 cd	Barely umami and mellow, slight harsh	88 ± 0.00c	Yellow-green	92 ± 0.50b	90.67 ± 0.11c

Note: Different letters in the same column indicate significant differences between fixation treatments (*p* < 0.05) based on the least significant difference test.

The heat radiation mode, microwave second-drying through high-frequency vibration, heated the tea leaves quickly, promoted the formation and retention of quality components, and the quality of green appearance with a clear floral aroma and strong and mellow taste. Therefore, microwave second-drying is the best method of heat radiation for green tea. The heat convection mode, with a long heating time, was not conducive to retaining chlorophyll from a green appearance. In the heat conduction mode, due to continuous friction between the leaves and the high-temperature pot wall, the cell fragmentation and leaching rate of tea leaves were higher. This effect promoted a sufficient thermal reaction and formed a higher quality aroma, but with a slightly bitter and astringent taste.

#### Appearance and liquor color properties

**Fig. 4:**
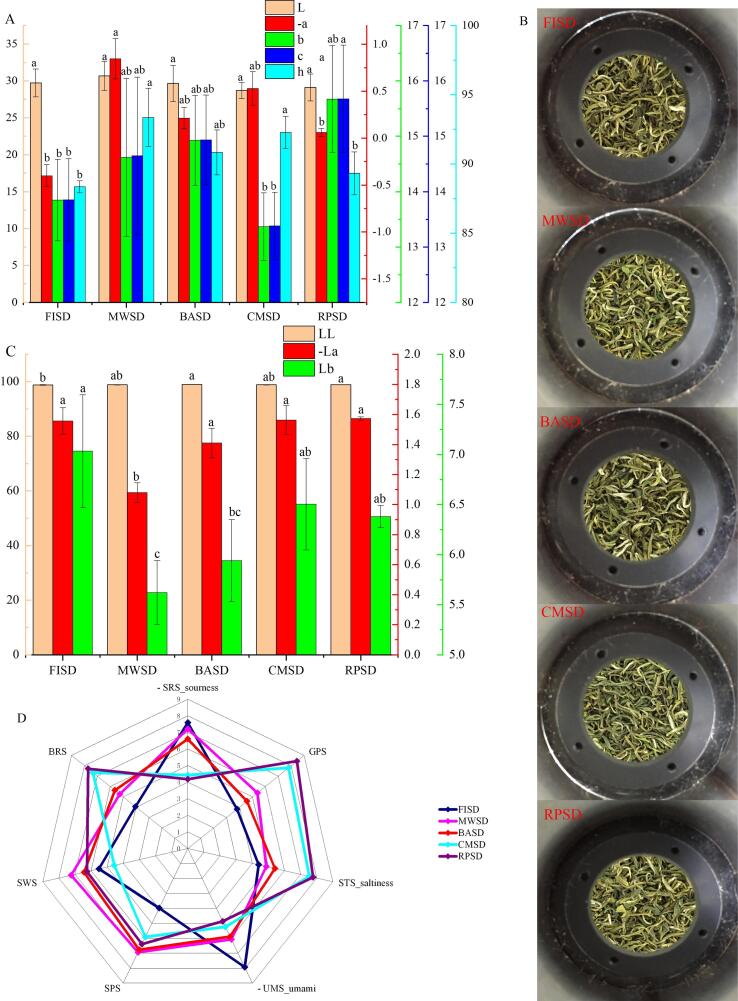
Effect of second-drying heat transfer methods on color attributes in green tea appearance (A-B) and soup (C). Effect of second-drying heat transfer methods on taste attributes (D). (For interpretation of the references to color in this figure legend, the reader is referred to the web version of this article.)

In terms of appearance color (Fig. 4A–B), there was no significant difference in the “L” value under different second-drying methods, while “-a” value was the highest in MWSD and the lowest in FISD and RPSD. “b”, “c”, and “h” values were significantly higher in MWSD than other treatments. In terms of soup color (Fig. 4C), the “LL” value was the highest in MWSD and RPSD and the lowest in FISD (*p* < 0.05); the “– La” value was the lowest under MWSD treatment (*p* < 0.05), while the “Lb” value was the highest in FISD. It was speculated that the low cell fragmentation rate and less water-soluble pigment leaching under microwave second-drying led to a shallow soup color, which corresponded to the results of the sensory evaluation. Combined with the results of the sensory evaluation, the quality of the green leaf, clear soup can be obtained under microwave treatment, which is the best method.

#### Taste properties

We used an electronic tongue with seven sensors, which included five taste attributes: acid (SRS), sweet (SWS), bitter (BRS), salty (STS), fresh (UMS), and two comprehensive attributes (GPS and SPS). The results are shown as a radar map (Fig. 4D). The electronic tongue response contour maps of CMSD, RPSD, FISD, and BASD were largely identical, but their intensities were different, while MWSD was significantly different (*p* < 0.05). Among them, CMSD and RPSD responded strongly to STS, BRS, and GPS, with values greater than seven, but weakly to UMS. The FISD responded strongly to SRS and UMS, and the MWSD responded strongly to SWS and SPS. That is, the sweetness, umami, and comprehensive attributes of green tea samples treated by heat radiation (microwave) and heat convection were higher, whereas the heat radiation (far-infrared) and heat conduction were lower, which is consistent with the artificial sensory results.

### Multivariate statistical analysis of the key quality differential metabolites at different heat transfer methods

#### Analysis of the key non-volatile quality differential compounds

**Fig. 5:**
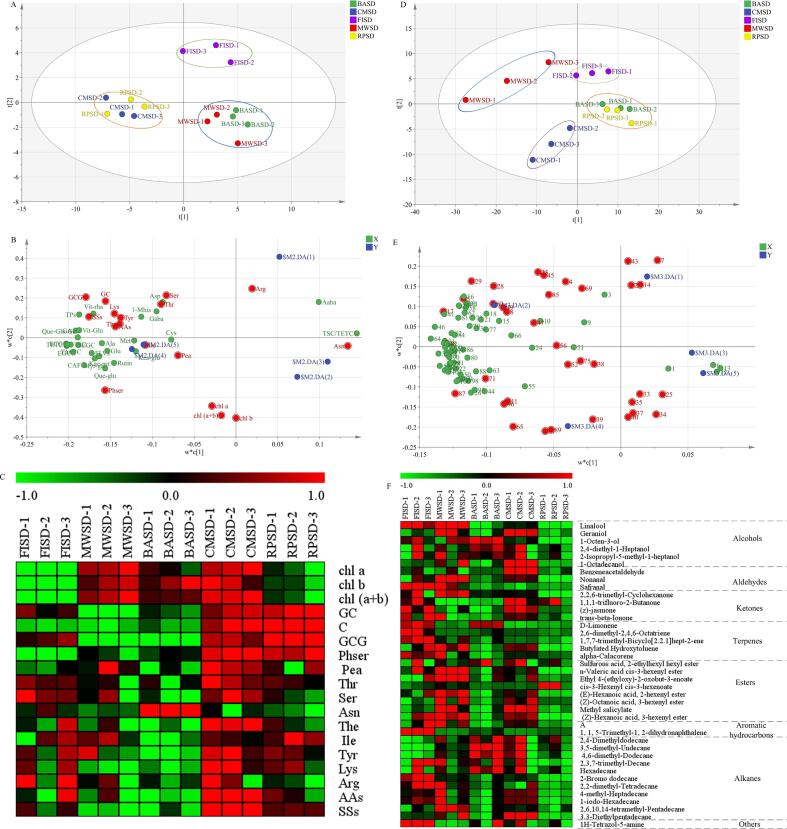
Multivariate statistical analysis of different second-drying methods: PLS-DA score (A), loading diagram (B), differential substance heat map (C) for non-volatile metabolites of green tea treated for different second-drying heat transfer methods. PLS-DA score (D), loading diagram (E), differential substance heat map (F) for volatile metabolites of green tea treated for different second-drying heat transfer methods. Note: The letter A in Figure E stands for (1S-cis)-1,2,3,5,6,8a-hexahydro-4,7-dimethyl-1-(1-methylethyl)-naphthalene. (For interpretation of the references to color in this figure legend, the reader is referred to the web version of this article.)

Based on the 45 non-volatile metabolites detected in green tea, PLS-DA was used to screen for key differences in metabolites that affect color and taste. As shown in Fig. 5A, the second-drying methods were distinguished (R2X = 0.846, R2Y = 0.93, Q2 = 0.728), the model had a strong cumulative interpretation and prediction ability, and is stable and reliable.

To further determine the key differences in nonvolatile metabolites, the variable projection importance (VIP) of the PLS-DA model was used for selection based on the principle of VIP > 1. As Fig. 5B shows, 17 non-volatile metabolites were identified, including chlorophyll *b*, chlorophyll (a + b), chlorophyll *a*, asparagine, GC, theanine, isoleucine, phosphor-serine, serine, arginine, total soluble sugar, tyrosine, phosphoethanolamine, threonine, total amino acids, lysine, and GCG. Among them, chlorophyll *b*, chlorophyll (a + b), and chlorophyll *a* were identified as the key substances that imparted the green appearance to green tea, GC and GCG were associated with the astringent taste and theanine, asparagine, arginine with the umami taste. Combined with the previous analysis, and the variation of the differential non-volatile compounds under different heat transfer methods is shown in the thermogram (Fig. 5C). Microwave second-drying was beneficial for the retention of chlorophyll *b*, chlorophyll (a + b), and chlorophyll *a*, and the appearance of the tea sample was greener. Heat conduction promoted the formation of GC, GCG and the retention of sugars and amino acids, which is not conducive to the formation of aroma, even taste with bitterness and astringency (Liu et al., 2014). Simultaneously, microwave second-drying promoted the formation and conversion of theanine, arginine, and total soluble sugars, and the aroma and taste were coordinated.

#### Analysis of the key volatile quality differential metabolites

##### PLS-DA

PLS-DA analysis was performed based on 101 volatile metabolites (Table S1). As shown in Fig. 5D, MWSD was located at the upper left of the plot, and CMSD was located at the bottom. FISD, BASD, and RPSD were located on the right side of the score map, FISD was located on the upper side, and BASD and RPSD were clustered on the lower side. This result is consistent with the sensory evaluation results; that is, the model clearly distinguished tea samples treated by different second-drying methods based on aroma type and aroma quality. The index of the model: R2X (cum) = 0.998, R2Y (cum) = 0.993, Q2 (cum) = 0.672, which demonstrates that the model had a strong cumulative interpretation and prediction ability, and is stable and reliable.

To further obtain the key differences in volatile metabolites, the VIP of the PLS-DA model was also used for selection based on the principle of VIP > 1. Twenty-nine important volatile metabolites (Fig. 5E), such as phenylacetaldehyde, linalool, d-limonene, methyl salicylate, geraniol, *trans*-β-ionone, nonanal, and (z)-jasmone were screened. The variation of the differential volatile compounds under different heat transfer methods is shown in the thermogram (Fig. 5F). Microwave second-drying was beneficial for the formation of linalool, geraniol, nonanal, safranal, (z)-jasmone, *trans*-β-ionone which give a flower and fruit fragrance that promoted the flowery aroma of the green tea.

##### OAV

OAV is an important indicator for objectively evaluating the contribution of volatile metabolites to the aroma. It is generally considered that substances with OAV > 1 contribute to the aroma, and those with OAV > 10 make important contributions to aroma (Zhu et al., 2021). In this study, the aroma thresholds and characteristics of ten volatile compounds (Table 3) were obtained by searching for references (Wang et al., 2020b, Wang et al., 2020d, Zhu et al., 2021) based on the 29 important volatile substances selected by PLS-DA analysis. There were 8 key volatile substances with OAV > 1, *trans*-β-ionone with OAV > 1900 was a key metabolite of green tea aroma; 1-octen-3-ol, linalool, nonanal, and (z)-jasmone, with OAV>10, were important metabolites; geraniol, benzeneacetaldehyde, and (z)-hexanoic acid, 3-hexenyl ester with OAV > 1 showed contribution to the aroma, among them, *trans*-β-ionone, (z)-jasmone, geraniol, and benzeneacetaldehyde have floral and fruity aromas, and were the main components of the pleasant aroma of green tea. The green tea under MWSD had the highest OAV values of 1-octen-3-ol, linalool, geraniol, nonanal, (z)-jasmone, *trans*-β-ionone, linalool, nonanal, and (z)-hexanoic acid-3-hexenyl ester (*p* < 0.05), so that the aroma quality was considered best. CMSD also had a high content of floral and fruity metabolites but also a high content of grassy metabolites (i.e., L-α-terpineol and cedarol), which lead to the formation of a grassy flavor. That is, microwave secondary drying was conducive to the formation of floral and fruity substances, and thus promoted a tender and clear floral fragrance.

**Table 3:** OAV values and aroma characteristics of key differences of volatile compounds treated with different second-drying methods.

Compounds name	OT (μg/L)^1^	Odor Characteristic^2^	FISD	MWSD	BASD	CMSD	RPSD
1-Octen-3-ol	1.5	Mushroom, Lavender, rose aroma	15.11 ± 3.33a	16.06 ± 1.72a	16.2 ± 5.84a	7.93 ± 0.53c	12.23 ± 0.23a
Linalool	6	Floral, lavender	9.43 ± 2.2b	12.29 ± 1.23a	7.32 ± 0.68bc	8.47 ± 0.2bc	6.94 ± 0.31c
Geraniol	40	Rose	0.24 ± 0.23c	1.19 ± 0.36a	0.08 ± 0.12c	0.79 ± 0.09b	0.19 ± 0.14c
Benzeneacetaldehyde	4	Woody, sweet aroma	0.42 ± 0.28b	1.77 ± 1.59ab	0.42 ± 0.16b	3.14 ± 1.82a	0.45 ± 0.12b
Nonanal	1	Sweet Orange aroma	10.42 ± 2b	16.58 ± 0.11a	9.48 ± 0.91bc	10.99 ± 1.4b	7.73 ± 0.38c
(z)-jasmone	0.26	Flowery	37.7 ± 12.6b	75.04 ± 25.21a	33.86 ± 11.18b	71.37 ± 7.38a	36.75 ± 7.65b
*trans*-β-Ionone	0.007	Violet-like, floral, and raspberry-like	1985.74 ± 292.73b	3248.34 ± 783.7a	2068.48 ± 164.81b	3136.41 ± 374.06a	2025.88 ± 333.37b
d-Limonene	10	Eucalyptus, lemongrass, citrus	0.84 ± 0.16a	0.54 ± 0.09bc	0.63 ± 0.07b	0.39 ± 0.02c	0.43 ± 0.03c
Methyl salicylate	40	Vanilla aroma	0.25 ± 0.03b	0.37 ± 0.09a	0.21 ± 0.07b	0.37 ± 0.03a	0.21 ± 0.04b
(z)-Hexanoic acid, 3-hexenyl ester	16	Fruity	3.33 ± 0.42b	4.87 ± 0.46a	2.89 ± 0.36 cd	3.78 ± 0.1b	2.61 ± 0.25d

Notes: Different lowercase letters in a row indicate a significant difference between the second-drying method (*p* < 0.05) based on the least significant difference test.

1 : Determined according tohttp://www.thegoodscentscompany.com/search3.php?qOdor=20126-76-5&submit.x=9&submit.y=9.

2 : OT, odor threshold in water based on the literature (Wang et al., 2020b, Wang et al., 2020d, Zhu et al., 2021).

## Conclusions

Heat transfer methods showed remarkable effects on the content of non-volatile and volatile metabolites, as well as the sensory quality and objective flavor indices, of green tea. MWSD can promote the degradation of flavonoid glycosides, the formation and transformation of amino acids and soluble sugars, and the retention of chlorophyll. The appearance greenness “-a” value and the umami, sweetness, and comprehensive attributes with electronic tongue were highest in MWSD. The MWSD tea samples were rich in high-boiling alcohols, aldehydes, and ketones, such as linalool, geraniol, *trans*-β-ionone, and (z)-jasmone, which promoted the formation of tender and floral aroma. The artificial sensory evaluation also showed the same results, with a yellow-green appearance, slightly yellow liquor color, tender and floral aroma, and a strong mellow taste. Using PLS-DA analysis, 17 key non-volatile compounds, including chlorophyll, asparagine, GC, theanine, isoleucine, serine, arginine, total sugar, threonine, lysine, and GCG, were obtained. Eight key volatile metabolites, including 1-octene-3-ol, linalool, phenylacetaldehyde, geraniol, nonanal, (z)-jasmone, (z)-hexanoic acid-hexenyl ester, and *trans*-β-ionone, were screened by PLS-DA and OAV analysis. These key metabolites can be used as an index for the directional and standardized processing of high-quality green tea. This study provides a theoretical basis and technical guidance for the precise and directional processing of high-quality green tea.

Due to the limitations of detection method, this study was unable to clarify the association between key non-volatile compounds and volatile compounds. In a future study, we hope will explore the influence of seconding-drying parameters on the evolution of key volatile and non-volatile compounds and the quality formation of green tea. Furthermore, we will perform an in-depth study on the formation mechanism of key quality components during the second-drying process.

## CRediT authorship contribution statement

**Huajie Wang:** Methodology, Conceptualization, Formal analysis, Investigation, Writing – original draft. **Wen Ouyang:** Data curation, Methodology, Investigation, Visualization. **Yaya Yu:** Data curation, Methodology, Software. **Jinjin Wang:** Investigation, Resources, Software. **Haibo Yuan:** Validation, Investigation, Writing – review & editing. **Jinjie Hua:** Conceptualization, Resources, Supervision, Writing – review & editing, Project administration. **Yongwen Jiang:** Supervision, Conceptualization, Resources, Project administration, Funding acquisition.

## Declaration of Competing Interest

The authors declare that they have no known competing financial interests or personal relationships that could have appeared to influence the work reported in this paper.
