# Supplementary material for: Analysis of non-volatile and volatile metabolites reveals the influence of second-drying heat transfer methods on green tea quality
Source: Food Chem X. 2022 Jun 3;14:100354. doi: 10.1016/j.fochx.2022.100354 (PMC9184872; doi:10.1016/j.fochx.2022.100354)
Supplement: Supplementary data 1 [file mmc1.docx]

**Analysis of non-volatile and volatile metabolites reveals the influence of second-drying heat transfer methods on green tea quality**

Huajie Wang^ab1^, Wen Ouyang^ac1^, Yaya Yu^a^, Jinjin Wang^a^, Haibo Yuan^a^, Jinjie Hua^a*^, Yongwen Jiang ^a*^

*^a^ Tea Research Institute, Chinese Academy of Agricultural Sciences, 9 Meiling South Road, Hangzhou, Zhejiang 310008, PR China*

*^b^ Institute of Tea Science, Zhejiang University, Hangzhou, 310058, P.R. China*

*^c^ State Key Laboratory of Tea Plant Biology and Utilization, Anhui Agricultural University, Hefei 230036, Anhui, P.R. China*

^1^ **These authors contributed equally to this work**

*******Corresponding authors:**

**Jinjie Hua**, Tea Research Institute, Chinese Academy of Agricultural Sciences, 9 Meiling South Road. Hangzhou, 310008, Zhejiang, PR China; Tel.: +86 15905819604/+86 0571-86650637; Fax number: +86 0571-85102505; Email: [huajinjie@tricaas.com](mailto:huajinjie@tricaas.com)

**Yongwen Jiang**, Tea Research Institute, Chinese Academy of Agricultural Sciences, 9 Meiling South Road, Hangzhou, 310008, Zhejiang, PR China; Tel.: +86 13957114526/+86 0571-86650103; Fax number: +86 0571-85102505; E-mail: jiangyw@tricaas.com

Table. S1 List all of volatile metabolites in the green tea samples made by different second-drying methods.

| NO | Name | RT | RI^A^ | RI^B^ | A1 | A2 | B | C1 | C2 |
| --- | --- | --- | --- | --- | --- | --- | --- | --- | --- |
| 2 | 1-Octen-3-ol | 11.30 | 996.48 | 986 | 22.67±5a | 24.1±2.58a | 24.31±8.76a | 11.89±1.29a | 18.34±0.34a |
| 15 | Ethyl 2-(5-methyl-5-vinyltetrahydrofuran-2-yl)propan-2-yl carbonate | 16.53 | 1143.60 | - | 7.47±1.53ab | 8.05±0.48a | 6.2±0.62b | 7.36±1.36ab | 6.21±0.07b |
| 16 | Linalool | 17.16 | 1161.50 | 1104 | 56.56±13.2b | 73.72±7.38a | 43.94±4.11bc | 50.82±1.2bc | 41.67±1.86c |
| 19 | (3R,6S)-2,2,6-Trimethyl-6-vinyltetrahydro-2H-pyran-3-ol | 20.78 | 1263.52 | 1183 | 2.68±0.66ab | 3.6±0.83a | 2.03±0.37b | 3.41±0.13a | 1.92±0.16b |
| 21 | L-.alpha.-Terpineol | 21.58 | 1285.96 | 1187 | 4.32±0.47ab | 5.6±2.23a | 2.68±0.41b | 4.24±1.27ab | 2.71±0.31b |
| 32 | Geraniol | 24.78 | 1375.89 | 1267 | 9.77±9.26c | 47.54±14.25a | 3.26±4.7c | 31.46±3.48b | 7.45±5.58c |
| 37 | 1-Heptanol, 2,4-diethyl- | 26.84 | 1433.99 | - | 0.86±0.38ab | 0.68±0.28b | 0.87±0.26ab | 1.29±0a | 0.94±0.25ab |
| 39 | 2-Isopropyl-5-methyl-1-heptanol | 27.58 | 1455.00 | - | 1.38±0.57b | 1.38±0.6b | 1.45±0.37b | 2.34±0.09a | 1.59±0.35ab |
| 72 | Nerolidol 2 | 37.67 | 1849.54 | - | 0.97±0.24c | 3.05±0.93a | 0.88±0.15c | 1.92±0.37b | 0.98±0.19c |
| 76 | Cedrol | 38.90 | 1971.77 | 1608 | 1.8±0.25b | 3.15±0.77a | 2.04±0.17b | 3.08±0.44a | 1.71±0.23b |
| 78 | Di-epi-1,10-cubenol | 39.97 | 2077.86 | 1623 | 3.07±0.32b | 6.72±2.1a | 2.65±0.22b | 5.66±1.24a | 2.88±0.55b |
| 79 | Epicubenol | 40.50 | 2129.81 | 1625 | 2.36±0.26b | 5.53±1.71a | 1.98±0.26b | 4.78±1.07a | 2.32±0.43b |
| 82 | 1-Hexadecanol | 40.91 | 2171.07 | 1883 | 7.92±0.54bc | 11.21±3.09a | 5.75±1.47c | 9.23±0.83ab | 5.14±0.67c |
| 95 | 1-Octadecanol | 48.16 | 2888.34 | 2090 | 1.53±0.05b | 3.64±1.4a | 1.35±0.28b | 3.06±0.6a | 1.25±0.23b |
| Alcohols | | | | | 123.38±32.75bc | 197.95±33.47a | 99.37±11.87c | 140.55±4.32b | 95.12±8.02c |
| 5 | Octanal | 12.42 | 1027.92 | 1004 | 1.06±0.2b | 2.03±0.34a | 0.59±0.36b | 0.86±0.14b | 0.43±0.58b |
| 11 | Benzeneacetaldehyde | 14.74 | 1093.36 | 1049 | 1.68±1.13b | 7.09±6.36ab | 1.69±0.65b | 12.54±7.29a | 1.79±0.46b |
| 17 | Nonanal | 17.37 | 1167.44 | 1102 | 10.42±2b | 16.58±0.11a | 9.48±0.91bc | 10.99±1.4b | 7.73±0.38c |
| 23 | 1,3-Cyclohexadiene-1-carboxaldehyde, 2,6,6-trimethyl- | 21.95 | 1296.23 | 1197 | 1.66±0.54a | 1.6±0.46a | 0.87±0.12b | 0.9±0.2b | 0.84±0.04b |
| 26 | Decanal | 22.37 | 1308.13 | 1200 | 1.07±0.15bc | 1.45±0.01a | 1.24±0.16b | 1.5±0.05a | 1.05±0.02c |
| 27 | 1-Cyclohexene-1-carboxaldehyde, 2,6,6-trimethyl- | 22.88 | 1322.66 | 1214 | 2.52±0.42b | 3.45±0.3a | 2.27±0.23b | 2.46±0.22b | 2.07±0.23b |
| 31 | 1-Cyclohexene-1-acetaldehyde, 2,6,6-trimethyl- | 24.59 | 1370.65 | 1254 | 0.8±0.16bc | 1.41±0.21a | 0.82±0.07bc | 1.05±0.02b | 0.74±0.11c |
| Aldehydes | | | | | 19.21±4.6b | 33.6±6.88a | 16.95±1.94b | 30.3±6.08a | 14.65±1.04b |
| 8 | Cyclohexanone, 2,2,6-trimethyl- | 13.76 | 1065.78 | - | 2.41±1.36b | 4.03±0.15a | 2.77±0.31b | 2.36±0.61b | 2.15±0.2b |
| 30 | 2,2-Dimethyl-3-octanone | 24.31 | 1362.76 | - | 0.75±0.2ab | 1.43±1.01a | 0.24±0.05b | 0.88±0.34ab | 0.28±0.01b |
| 50 | 2-Cyclopenten-1-one, 3-methyl-2-(2-pentenyl)-, (Z)- | 31.06 | 1552.87 | 1396 | 9.8±3.28b | 19.51±6.55a | 8.8±2.91b | 18.56±1.92a | 9.55±1.99b |
| 51 | 2-Butanone, 1,1,1-trifluoro- | 31.69 | 1570.62 | - | 2.01±0.38a | 1.54±0.84a | 0.89±0.02a | 2±0.88a | 1.47±0.82a |
| 54 | 5,9-Undecadien-2-one, 6,10-dimethyl-, (E)- | 33.27 | 1615.09 | 1460 | 3.03±0.44b | 6.62±1.22a | 3.06±0.55b | 5.54±0.42a | 3±0.71b |
| 59 | trans-.beta.-Ionone | 34.55 | 1651.23 | 1498 | 13.9±2.05b | 22.74±5.49a | 14.48±1.15b | 21.95±2.62a | 14.18±2.33b |
| 61 | 3-Tridecanone | 34.83 | 1659.10 | 1498 | 4.51±0.13b | 6.64±0.84a | 3.15±0.68c | 4.12±0.74bc | 3.97±0.72bc |
| Ketones | | | | | 36.42±7.44b | 62.52±15.79a | 33.39±3.88b | 55.41±4.54a | 34.61±5.95b |
| 20 | Butanoic acid, 3-hexenyl ester, (E)- | 21.46 | 1282.43 | 1185 | 9.84±2.15b | 13.16±0.21a | 8.66±1.36bc | 9.64±0.97bc | 7.45±0.56c |
| 22 | Methyl salicylate | 21.89 | 1294.52 | 1197 | 9.9±1.01b | 14.98±3.45a | 8.33±2.65b | 14.8±1.05a | 8.44±1.61b |
| 25 | Sulfurous acid, 2-ethylhexyl hexyl ester | 22.19 | 1303.06 | - | 2±0.59ab | 1.45±0.46b | 1.85±0.5ab | 2.45±0.4a | 2.13±0.71ab |
| 29 | n-Valeric acid cis-3-hexenyl ester | 23.60 | 1342.86 | 1236 | 2.05±0.31b | 2.61±0.11a | 1.62±0.28c | 1.65±0.11c | 1.33±0.08c |
| 41 | (E)-Hex-3-enyl (E)-2-methylbut-2-enoate | 27.94 | 1465.13 | - | 0.65±0.09b | 1.02±0.2a | 0.38±0.14c | 0.67±0.04b | 0.37±0.08c |
| 46 | Hexanoic acid, 3-hexenyl ester, (Z)- | 30.23 | 1529.55 | 1381 | 53.3±6.76bc | 77.88±7.33a | 46.27±5.8cd | 60.46±1.57b | 41.74±4.02d |
| 47 | cis-3-Hexenyl cis-3-hexenoate | 30.46 | 1535.87 | 1389 | 2.61±0.95b | 27.42±20.4a | 2.86±0.46b | 2.74±0.03b | 12.06±17.02ab |
| 48 | Hexanoic acid, 2-hexenyl ester, (E)- | 30.62 | 1540.42 | 1391 | 6.8±0.91c | 13.92±1.89a | 7.46±1.41bc | 9.64±0.38b | 7.11±0.8c |
| 52 | Carbonic acid, octadecyl vinyl ester | 31.86 | 1575.38 | - | 3.18±0.9a | 2.85±1.04a | 2.55±0.75a | 3.59±0.71a | 2.51±0.56a |
| 74 | Octanoic acid, 3-hexenyl ester, (Z)- | 38.24 | 1905.83 | - | 3.18±0.49c | 6.82±1.48a | 3.18±0.49c | 5.03±0.86b | 3.26±0.46c |
| 91 | Carbonic acid, eicosyl vinyl ester | 45.72 | 2646.57 | - | 0.89±0.09b | 2.6±1.17a | 0.86±0.27b | 2.07±0.38a | 0.77±0.12b |
| 96 | Phthalic acid, hept-4-yl isobutyl ester | 48.82 | 2953.64 | - | 3.17±2.44b | 12.99±10.1ab | 7.38±3.25b | 20.22±4.62a | 3.06±5.12b |
| 98 | Hexadecanoic acid, methyl ester | 50.66 | 3135.90 | - | 0.38±0.29b | 2.37±1.35a | 0.62±0.18b | 2.13±0.58a | 0.7±0.13b |
| 99 | Benzenepropanoic acid, 3,5-bis(1,1-dimethylethyl)-4-hydroxy-, methyl ester | 50.99 | 3168.40 | - | 0.47±0.14b | 1.96±0.91a | 0.49±0.06b | 1.87±0.56a | 0.51±0.15b |
| 100 | Hexadecanoic acid, ethyl ester | 52.82 | 3349.84 | - | 0.97±0.34b | 3.22±1.32a | 0.88±0.33b | 3.29±0.92a | 0.83±0.07b |
| Esters | | | | | 99.38±11.31c | 185.26±10.22a | 93.39±15.84c | 140.24±8.11a | 92.25±19.27c |
| 4 | .beta.-Myrcene | 11.66 | 1006.65 | 981 | 6.86±2.19a | 6.32±0.76ab | 5.39±0.72ab | 5±0.24ab | 4.42±0.4b |
| 7 | D-Limonene | 13.41 | 1055.72 | 1030 | 8.38±1.64a | 5.41±0.92bc | 6.31±0.66b | 3.88±0.19c | 4.26±0.34c |
| 9 | Bicyclo[3.1.1]hept-2-ene, 3,6,6-trimethyl- | 14.06 | 1074.09 | - | 2.23±1.56a | 2.55±0.54a | 2.44±0.36a | 1.99±0a | 1.78±0.14a |
| 10 | .beta.-Ocimene | 14.55 | 1088.02 | 1037 | 5.01±3.75a | 7.18±0.24a | 5.7±0.94a | 4.86±0.24a | 4.32±0.17a |
| 14 | 2,4,6-Octatriene, 2,6-dimethyl- | 16.46 | 1141.70 | 1147 | 2.78±1.79a | 0.84±0.2b | 0.91±0.24b | 0.78±0.01b | 0.6±0.1b |
| 18 | (E)-4,8-Dimethylnona-1,3,7-triene | 17.93 | 1183.19 | 1089 | 4.72±1.84ab | 6.42±0.72a | 4.37±0.92b | 4.69±0.51ab | 3.67±0.14b |
| 28 | Bicyclo[2.2.1]hept-2-ene, 1,7,7-trimethyl- | 23.27 | 1333.61 | - | 1.18±0.15ab | 1.28±0.35a | 0.71±0.12c | 0.88±0.15bc | 0.62±0.09c |
| 42 | .alpha.-Cubebene | 28.68 | 1485.81 | 1354 | 4.85±2.32c | 16.19±3.37a | 6.6±0.82c | 12.61±0.75b | 6.19±0.75c |
| 43 | Bicyclosesquiphellandrene | 34.61 | 1652.81 | 1521 | 1.96±1.15bc | 6.45±2.3a | 2.08±0.32bc | 4±0.87b | 1.77±0.16c |
| 60 | .alpha.-Muurolene | 35.00 | 1663.84 | 1505 | 3.89±0.07a | 5±1.58a | 2.28±0.34c | 3.72±0.6ab | 2.36±0.27bc |
| 64 | Cubenene | 36.23 | 1707.52 | 1552 | 9.7±6.78a | 5.19±1.29ab | 2.08±0.34b | 3.97±0.36b | 2.08±0.27b |
| 69 | .alpha.-Calacorene | 36.68 | 1751.32 | 1542 | 10.66±0.77abc | 14.01±5a | 6.15±0.87c | 11.2±2.79ab | 6.75±1.31bc |
| 70 | .tau.-Muurolol | 41.03 | 2182.20 | 1640 | 0.96±0.17b | 2.6±0.86a | 0.69±0.08b | 2.27±0.63a | 0.84±0.19b |
| 83 | Neophytadiene | 47.50 | 2822.90 | 1806 | 0.21±0b | 2.64±1.65a | 0.32±0.04b | 3±1.55a | 0.46±0.16b |
| Terpenes | | | | | 63.39±5.51b | 82.09±18.34a | 46.05±6.37c | 62.85±6.53b | 40.1±3.89c |
| 53 | (1S,4S,4aS)-1-Isopropyl-4,7-dimethyl-1,2,3,4,4a,5-hexahydronaphthalene | 32.92 | 1605.32 | 1458 | 1.82±1.32b | 6.31±2.38a | 1.91±0.37b | 3.95±0.8b | 1.71±0.24b |
| 57 | 1-Isopropyl-4,7-dimethyl-1,2,3,4,5,6-hexahydronaphthalene | 33.90 | 1632.78 | 1481 | 3.15±1.71b | 9.28±3.07a | 3.06±0.73b | 6.29±1.09b | 3.07±0.53b |
| 58 | Naphthalene, 1,2,3,4,4a,5,6,8a-octahydro-7-methyl-4-methylene-1-(1-methylethyl)-, (1.alpha.,4a.beta.,8a.alpha.)- | 34.03 | 1636.42 | 1511 | 1.68±0.16a | 2±0.48a | 1.07±0.16b | 1.56±0.17a | 1.04±0.12b |
| 65 | Phenol, 2,5-bis(1,1-dimethylethyl)- | 35.64 | 1649.15 | - | 0.79±0.25c | 1.71±0.3b | 1.32±0.24bc | 2.91±0.64a | 1.05±0.23bc |
| 67 | Naphthalene, 1,2,3,4-tetrahydro-1,6-dimethyl-4-(1-methylethyl)-, (1S-cis)- | 35.91 | 1675.23 | 1521 | 56.54±12.21a | 57.3±14.19a | 28.77±3.72c | 47.49±7.52ab | 31.54±6.25bc |
| 68 | Naphthalene, 1,2,3,5,6,8a-hexahydro-4,7-dimethyl-1-(1-methylethyl)-, (1S-cis)- | 35.93 | 1677.45 | 1524 | 20.31±3.84bc | 40.38±11.34a | 15.57±2.77c | 29.52±4.5b | 16.3±2.09c |
| 62 | 1, 1, 5-Trimethyl-1, 2-dihydronaphthalene | 28.79 | 1488.89 | - | 2.18±0.1a | 0.91±0.4b | 0.7±0.14b | 0.57±0.12b | 0.57±0.09b |
| 85 | Naphthalene, 1,6-dimethyl-4-(1-methylethyl)- | 41.72 | 2250.48 | 1684 | 4.23±2.08a | 3.25±1.13ab | 1.48±0.19b | 2.61±0.51ab | 1.7±0.59b |
| 93 | Butylated Hydroxytoluene | 35.55 | 1639.41 | 1533 | 2.07±0.26c | 3.38±0.39a | 1.84±0.21cd | 2.65±0.12b | 1.62±0.15d |
| Aromatic hydrocarbons | | | | | 92.76±6.65b | 124.5±33.68a | 55.71±8.46c | 97.55±15.46b | 58.6±9.6c |
| 1 | Heptane, 2,4-dimethyl- | 4.50 | 805.03 | - | 1.73±1.51a | 0.9±0.3a | 1.87±0.63a | 1.94±0.69a | 1.43±0.99a |
| 6 | Octane, 3,3-dimethyl- | 13.20 | 1049.93 | - | 1.64±1.27a | 0.78±0.18a | 1.73±0.53a | 1.56±0.7a | 1.73±0.53a |
| 12 | Nonane, 4,5-dimethyl- | 14.96 | 1099.38 | - | 10.76±7.42a | 5.52±1.33a | 11.24±2.64a | 10.86±4.37a | 12.21±3.5a |
| 13 | 3-Ethyl-3-methylheptane | 15.22 | 1106.89 | - | 2.25±1.57a | 1.06±0.16a | 2.3±0.61a | 2.17±0.89a | 2.45±0.67a |
| 24 | Dodecane | 21.97 | 1296.85 | - | 4.44±1.39ab | 4.52±0.55ab | 4.2±0.83ab | 4.82±0.21a | 3.24±0.18b |
| 33 | 2,4-Dimethyldodecane | 24.94 | 1380.66 | - | 4.48±1.77ab | 3.06±1.41b | 3.91±1.26ab | 5.6±0.32a | 4.1±0.59ab |
| 34 | Undecane, 3,5-dimethyl- | 26.06 | 1412.00 | - | 2.5±0.87ab | 1.86±0.68b | 2.83±0.96ab | 3.7±0.1a | 2.71±0.7ab |
| 35 | Dodecane, 4,6-dimethyl- | 26.31 | 1419.19 | - | 3.55±1.2ab | 2.68±0.77b | 3.53±1.12ab | 4.71±0.09a | 3.25±0.68ab |
| 36 | 1-Oxaspiro[4.5]dec-6-ene, 2,6,10,10-tetramethyl- | 26.37 | 1420.75 | 1303 | 2.53±0.22b | 3.88±1.35a | 1.44±0.25b | 2.18±0.58b | 1.31±0.23b |
| 38 | Decane, 2,3,7-trimethyl- | 26.97 | 1437.59 | - | 1.59±0.52a | 1.2±0.53a | 1.18±0.62a | 1.74±0.06a | 1.16±0.22a |
| 40 | Hexadecane | 27.75 | 1459.54 | - | 7.25±2.83b | 5.83±2.38b | 7.82±2.7ab | 11.53±1.08a | 7.81±1.75ab |
| 44 | Undecane, 4,4-dimethyl- | 28.94 | 1493.25 | - | 0.81±0b | 1.6±0.45a | 1.08±0.33ab | 1.61±0.33a | 0.98±0.11b |
| 45 | 2-Bromo dodecane | 29.67 | 1513.82 | - | 3.08±0.72a | 2.31±0.51ab | 1.37±0.4c | 1.74±0.02bc | 1.02±0.06c |
| 49 | Tetradecane | 30.97 | 1550.25 | - | 8.39±2.45a | 8.16±2.02a | 5.14±1.05bc | 7.24±0.67ab | 4.24±0.12c |
| 56 | 5,5-Dibutylnonane | 33.62 | 1625.06 | - | 1.54±0.22a | 1.2±0.5ab | 1.03±0.14b | 1.54±0.07a | 0.96±0.09b |
| 63 | Pentadecane | 35.07 | 1665.67 | - | 4.31±0.82ab | 5.13±1.74ab | 3.41±0.69b | 5.74±0.8a | 3.41±0.07b |
| 66 | Tetradecane, 2,2-dimethyl- | 35.67 | 1651.48 | 1515 | 6.28±1.02a | 7.47±2.11a | 3.98±0.85a | 6.78±5.38a | 3.28±0.28a |
| 73 | Pentadecane, 3-methyl- | 37.82 | 1864.55 | 1570 | 5.01±0.64a | 6.01±1.9a | 3.08±1.1bc | 4.9±0.13ab | 2.8±0.56c |
| 75 | 2-Ethylthiolane, S,S-dioxide | 38.67 | 1949.19 | - | 0.37±0.17c | 0.89±0.01a | 0.82±0.15ab | 0.58±0.09bc | 0.7±0.24ab |
| 77 | Azetidine | 39.54 | 2035.18 | - | 1.55±0.33ab | 1.76±0.7a | 0.91±0.31bc | 1.61±0.36ab | 0.8±0.08c |
| 80 | Undecane, 6-ethyl- | 40.67 | 2146.58 | - | 1.18±0.13ab | 1.73±0.66a | 0.8±0.35b | 1.68±0.28a | 0.82±0.19b |
| 81 | 3,3-Diethyltridecane | 40.80 | 2159.86 | 1653 | 1.34±0.1a | 1.72±0.52a | 0.79±0.24b | 1.35±0.07a | 0.72±0.07b |
| 84 | Heptadecane, 4-methyl- | 41.38 | 2216.99 | - | 0.31±0.02bc | 0.5±0.18a | 0.23±0.07c | 0.39±0.04ab | 0.2±0.03c |
| 86 | Hexadecane, 1-iodo- | 42.38 | 2316.63 | - | 3.08±0.22ab | 4.18±1.41a | 1.92±0.68b | 4.06±0.94a | 1.76±0.25b |
| 87 | Heptadecane | 42.66 | 2344.01 | - | 2.51±0.17b | 6.89±1.15a | 3.01±0.94b | 7.16±1.09a | 3.18±0.94b |
| 88 | Pentadecane, 2,6,10,14-tetramethyl- | 42.86 | 2364.05 | 1703 | 4.47±0.63c | 7.37±1.41a | 4.91±1.25bc | 6.93±2.07ab | 4.3±0.6c |
| 89 | Heneicosane | 43.00 | 2377.09 | - | 5.72±1.32b | 9.1±4.21b | 7.26±1.69b | 14.82±2.11a | 9.22±2.64b |
| 90 | Heptadecane, 3-methyl- | 45.16 | 2591.28 | 1771 | 0.35±0.01b | 0.71±0.24a | 0.31±0.08b | 0.59±0.09a | 0.28±0.04b |
| 92 | 5,5-Diethylpentadecane | 46.24 | 2698.54 | 1805 | 0.89±0.04bc | 1.7±0.6a | 0.65±0.2c | 1.31±0.17ab | 0.58±0.08c |
| 94 | 3,3-Diethylpentadecane | 47.99 | 2871.53 | - | 0.36±0.01b | 0.82±0.3a | 0.29±0.08b | 0.65±0.11a | 0.27±0.05b |
| 97 | Dotriacontane, 1-iodo- | 50.27 | 3097.13 | - | 1.23±0.26c | 2.81±1.64b | 1.74±0.3bc | 4.63±0.38a | 2.94±0.76b |
| 101 | 5,5-Diethylheptadecane | 52.85 | 3352.52 | - | 0.55±0.03b | 1.84±0.66a | 0.59±0.22b | 1.58±0.3a | 0.56±0.08a |
| Alkanes | | | | | 96.06±29.85ab | 105.18±32.53ab | 85.37±17.27b | 127.7±10.6a | 84.41±9.68b |
| 3 | Pentanoic acid, 2-methyl-, anhydride | 11.46 | 1000.91 | - | 1.42±0.76a | 1.33±0.09a | 1.1±0.17a | 0.94±0.41a | 1.1±0.06a |
| 55 | 1H-Tetrazol-5-amine | 33.52 | 1622.13 | - | 2.37±0.66ab | 2.79±0.88ab | 2.09±0.73b | 3.43±0.71a | 2.18±0.52ab |
| 71 | 1,2-Benzenediol, O-(1-naphthoyl)- | 37.02 | 1785.21 | - | 6.24±0.04bc | 7.94±2.95ab | 3.78±0.55c | 9.4±1.4a | 5.38±1.3bc |
| Others | | | | | 10.02±1.46ab | 12.06±3.92ab | 6.97±1.21c | 13.77±1.7a | 8.67±1.6bc |
| Total content of compounds | | | | | 540.61±86.26bc | 803.17±154.83a | 437.22±64.67c | 668.37±48.7ab | 428.41±41.49c |
